# A case-based complexity approach to health inequality: Understanding and tracing place-based differences to enhance policy calibration

**DOI:** 10.1016/j.ssmph.2026.101903

**Published:** 2026-01-27

**Authors:** Brian Castellani, Jonathan Wistow

**Affiliations:** a Department of Sociology, Durham Research Methods Centre, Wolfson Research Institute for Health and Well-being, Durham University, United Kingdom

## Abstract

Health inequalities are not static gradients of deprivation but emergent properties of complex, place-based social systems. This study applied a case-based complexity (CBC) approach, via the COMPLEX-IT platform, to analyse healthy life expectancy (HLE) in 141 English local authorities. The power of CBC lies in moving beyond aggregate-level conclusions to a trajectory-based analysis that captures the configurational dynamics of health inequality. Rather than treating disparities as linear outcomes of deprivation, CBC identifies cluster-specific patterns, offering a more precise policy intervention framework. These clusters are interpreted as *traces of complex systems*, offering a basis for investigating how socio-spatial processes shape health inequalities over time. We argue that reducing health inequalities requires a shift away from narrowly targeted interventions toward configurational-informed, multi-level governance. This includes recognising the interdependence of places, anticipating cross-cluster effects, and embedding adaptive feedback mechanisms in policy design. The paper also develops a CBC rubric to build on and enhance the analysis provided here. In so doing, our framework also supports a proportionate universalism that is locally calibrated while systemically coherent. By combining a complexity-informed, configurational-based, machine learning set of methods, this paper demonstrates how CBC is a conceptual and methodological advance on policy-relevant approaches for addressing persistent and embedded health inequalities across place.

## Highlights

• Health inequalities are traces of complex, place-based social systems.
• CBC reveals HLE trajectories shaped by local system configurations.
• Patterns emerge within England's austerity-constrained policy context.
• Clusters reflect relational inequalities, not simple deprivation gradients.
• Proportionate universalism needs local calibration and systemic coherence.

## 1 Introduction

Health inequalities remain one of the most persistent (Scambler & Scambler, 2015) and systemic challenges in public policy, governance, and public health (Wistow, 2022). These disparities are not simply variations in individual health outcomes but are deeply embedded in the social, economic, and institutional trajectories of place, reflecting the compounded effects of policy decisions, economic restructuring, and social stratification over time. Despite extensive research, dominant approaches continue to rely on reductionist, variable-driven models that fail to account for the complex, context-dependent interactions shaping health outcomes (Doyal and with Pennell, 1979; Navarro, 2009; Schrecker, 2017). As Salway and Green (2017) argue, public policy often misidentifies multi-level system boundaries, failing to extend causal analysis far enough up the chain to capture the higher-order political, economic, and governance structures that shape place-based health inequalities. Following Wistow et al. (2015), we will argue that health inequalities should be analysed as emergent properties of place-based social complexities: nonlinear, historically contingent and evolving configurations of social determinants, governance structures, and policy responses. In support of this argument, we will employ a *case-based complexity approach* to disrupt conventional understandings of health inequalities (Castellani & Gerrits, 2024).

### 1.1 Case-based complexity

For *case-based complexity* (CBC) scholars (e.g., Byrne & Ragin, 2009; Castellani & Gerrits, 2024), addressing complex phenomena like health inequalities begins with understanding how they take shape: through the evolving configurations of social, institutional, environmental, and historical forces that gather in and through cases, giving rise to the place-shaped trajectories along which disparities emerge and transform. Taking this view as foundational, CBC employs methods that trace or model these different patterned trajectories (i.e., major and minor cluster trends) as they unfold across time and place, revealing how inequalities crystallise through specific configurations rather than through isolated factors. From this follows a configurational view of policy: that meaningful policy interventions engage and align with these major and minor cluster trends as they are lived and situated, working with the systemic entanglements through which inequalities develop. Grounded in complex realism and configurational analysis (Byrne & Ragin, 2009), CBC offers a way of orienting policy to the dynamics of inequality, treating intervention as the practice of working with trajectories of inequality as they unfold across time and place (e.g., Castellani et al., 2015; Gerrits et al., 2021; Pearce, 2018).

COMPLEX-IT is one of a handful of platforms that give case-based complexity its methodological form (Gerrits et al., 2021; Schimpf & Castellani, 2020). COMPLEX-IT is an interdisciplinary methods platform for applied social inquiry and policy evaluation, designed to increase non-expert access to the tools of computational social science (Schimpf & Castellani, 2024). Its freely available R-Studio/Shiny software package is comprised of a bespoke suite of techniques, including cluster analysis, artificial intelligence, data visualization, data forecasting, case-based systems mapping, and case-based scenario simulation – collectively referred to as *case-based computational modelling* (Castellani et al., 2016). Having been used across a variety of public policy environments (Barbrook-Johnson & Penn, 2022; Castellani et al. 2019, 2025), COMPLEX-IT enables researchers, analysts and policymakers to (1) cluster cases according to their different profiles of factors relative to some outcome of concern; (2) create and explore each cluster's causal maps; (3) model and forecast these clusters and their trends across time; and (4) simulate, in a failure-safe environment, different policy interventions and their outcomes (Schimpf & Castellani, 2020).

Taken together, CBC and COMPLEX-IT provide a complexity-appropriate, practice-ready framework for understanding health inequalities as emergent, multi-pathway, place-based phenomena. They offer policymakers the conceptual and analytic means to see, and intervene in, the configurational dynamics through which inequalities take shape.

### 1.2 Policy, place and time

The Marmot Reviews (2010 and 2020a) and World Health Organisation (2008 and 2025) have contributed to the development of a social determinants of health (SDH) perspective and emphasise the significance of these for a social gradient in health. Globally, the SDH include: income and social protection; education; unemployment and job insecurity; working life conditions; food insecurity; housing, basic amenities and the environment; early child development; social inclusion and non-discrimination; structural conflict; and access to affordable health service of decent quality, and how these influence health equity in positive and negative ways (WHO, n.d.). The WHO (n.d.) continues to describe these as, ‘a set of forces and systems shaping the conditions of daily life … [and] … include economic policies, and systems, development agendas, social norms, social policies and political systems.’ This is a suitably broad definition, and one that aligns well with a complexity-informed frame of reference (Byrne & Callaghan, 2023), given the emergent, interconnected, and interdisciplinary nature of the system-oriented forces and dynamics at play. A key factor in understanding whether the SDH function as pathogenic or protective mechanisms relates to the socio-economic characteristics and positionality of individuals and places – namely, the social gradient. In this respect, Marmot et al., (2010) describe the SDH as the ‘causes of the causes’ of health inequalities, i.e., they shape/cause the behaviours (e.g., smoking, exercise, and diet), which are in turn causes associated with non-communicable diseases like cancers and CVD and outcomes such as HLE. By integrating a multi-level conceptualisation of place-based social complexity and aligning this with the established SDH evidence base (e.g., Marmot et al., 2020a; Marmot et al., 2020b) alongside its wider sociopolitical implications (Scambler & Scambler, 2015), this paper seeks to engage directly with a persistent blind spot within social complexity scholarship – the under-theorised role of power and inequality in shaping societal outcomes, as identified by Castellani and Gerrits (2024).

The SDH evidence base is an extremely useful resource for those concerned with health inequalities and health equity. However, we wish to highlight two somewhat contradictory issues that have undermined the efficacy of this, from a complexity-informed approach to calibrating policy to place-based differences in health. Firstly, despite the widespread reach of SDH across both academic and policy discourse the redistributive implications of the agenda (i.e., to narrow the social gradient in health) are underdeveloped (Wistow, 2022) and policy levers such as taxation have been marginal when considering forms of state intervention in this field (Lynch, 2017). Secondly, despite the Marmot Reviews (2010 and 2020a) highlighting the significance of local governance and policy systems adapting good practice to local contexts there has been a degree of determinism associated with the health inequalities and SDH agenda. Through a complexities-of-place lens, this paper seeks to move beyond the predictable narrative that poverty correlates with poor health to reveal the deeper, systemic patterns shaping these disparities. In this respect, focusing on place over time and considering trajectories of these in configurational terms advances an approach to understanding health inequalities outcomes as being path *dependent,* as opposed to *determined*.

We therefore situate this study within the wider international and interdisciplinary evidence base (as developed by, for example, Marmot et al., 2010; WHO 2025) that underpins the SDH perspective, while explicitly arguing for a stronger integration of insights from economic geography (Martin et al., 2021), political economy (Streeck, 2016), critical public health (Schrecker, 2021), and complexity theory (Moore et al., 2019). In doing so, we position our approach as a means of bridging these literatures to address a persistent gap in the field (Navarro, 2009; Scambler & Scambler, 2015): the need for analytical frameworks that can account for the spatial, political, economic and systemic dynamics through which health inequalities are produced and sustained. Taken together, this positioning underscores the value of a case-based complexity framework as a means of operationalising these interdisciplinary insights, enabling us to trace how broader structural forces become manifest in the specific configurations and trajectories observed across place.

Finally, the trajectory of health outcomes in England has international significance given the programme of austerity between 2010 and 2020 and how this is associated with stagnant (Marmot et al., 2020a) and now deteriorating life expectancy and HLE outcomes (Raleigh, 2025) at a national level. The austerity programme took precedence over all other measures in the 2010 UK Government Coalition Agreement (Cabinet Office, 2010) and has had a negative impact on social outcomes in England, including exacerbating inequalities in these (see, for example, UN, 2019; Marmot et al., 2020a). As Lansley (2021) has argued, this forms part of a longer-term, state-driven shift away from the post-war commitment to collective welfare and redistribution, and towards market-led ideologies associated with thinkers such as Hayek and Friedman. The dismantling of more egalitarian policy frameworks has not only contributed to the growing concentration of wealth among the super-rich and the widening of social and economic inequalities but has also occurred alongside a period of persistently low economic growth over the past 40+ years (Lansley, 2021; Streeck, 2016).

### 1.3 England health inequalities case study

To demonstrate the value of our approach, we applied CBC and the COMPLEX-IT platform to a health inequalities dataset comprised of 141 ‘upper tier’ local authorities in England,^1^ each characterized by distinct configurations of healthy life expectancy trends, social determinants, indices of deprivation, and policy-related preventable health outcomes. We chose this example for several reasons. First, England provides an instructive case study, given its longstanding focus on health inequalities (Acheson, 1998; Black, 1980; Marmot et al., 2010; 2020a), combined with extensive national health data availability.

Second, the policy landscape, shaped by reports like *Health Equity in England: The Marmot Review 10 Years On*, has consistently emphasized the importance of socioeconomic determinants in shaping health outcomes. Despite this people are dying earlier and spending more time in poor health with intersecting inequalities across deprivation, race, ethnic group and sex (Haim et al., 2024). Austerity-era cuts disproportionately affected prevention and wider social determinant services compared to treatment-based care (Marmot, 2020a), contributing to a developing public health crisis (Hunter et al., 2024).

Third, England's health interventions remain highly centralised and often lack the spatial granularity needed to address disparities at the local level (Childs et al., 2024; UK2070 Commission, 2020; Redhead & Lynch, 2024). In this respect, Marmot's (2015) principle of proportionate universalism, allocating resources based on level of need, holds significant promise. However, a critical research question follows: *how proportionate should proportionate universalism be?* Addressing this is complex, requiring careful calibration, alignment with local context, and sustained resource investment to ensure equitable and effective implementation.

Finally, our aim was not to restate the well-known link between deprivation and poor health, but to examine how a range of place-based characteristics of relevance to the health inequalities evidence-base configure over time. To do that, we needed to create a somewhat different dataset. As shown in Table 1, we chose social determinants, deprivation indices and health outcomes that effective policy and service provision should prevent or, at minimum, reduce. These indicators offer a practical lens on system performance rather than structural disadvantage alone. As a case study, while the UK provides an unusually rich administrative dataset, the same configurational logic can be adapted elsewhere for other countries using a smaller set of proxy indicators that capture preventable outcomes and the core social conditions policy is meant to address. In sum, this case study aims to show how applying CBC allows policy, place and time to become central to this equation, moving beyond aggregate-level conclusions to identify meaningful local authority-specific trends that can be used to inform more precise policy interventions.

**Table 1:** Cluster profiles of indicators: Local authority-level Configurations.

CLUSTER MEMBERSHIP FOR N-141 UK LOCAL AUTHORITIES	CLUSTER 1 Resilient Affluence	CLUSTER 2 Stagnant Stability	CLUSTER 3 Struggling to Hold Ground	CLUSTER 4 Declining Periphery	CLUSTER 5 Entrenched Disadvantage
***Number of Cases***	*53.00*	*13.00*	*12.00*	*15.00*	*48.00*
***NAME***	Mean and standard deviation (SD) reported for all HLE trends and for all N = 41 factors.				
Weighted Healthy Life Expectancy 2011-2013	66.18 SD 1.91	63.36 SD 1.20	63.12 SD 1.10	62.13 SD 1.50	58.99 SD 1.66
Weighted Healthy Life Expectancy 2013-2015	66.72 SD 1.90	63.78 SD 1.58	63.27 SD .87	62.03 SD .78	59.12 SD 1.50
Weighted Healthy Life Expectancy 2015-2017	66.46 SD 1.81	63.88 SD .92	62.71 SD 1.00	62.29 SD .87	59.34 SD 1.43
Weighted Healthy Life Expectancy 2017-2019	66.34 SD 1.80	63.00 SD 1.12	62.78 SD 1.11	62.10 SD .64	59.41 SD 1.57
Weighted Healthy Life Expectancy 2019-2021	66.32 SD 2.08	63.68 SD .84	62.51 SD .99	62.16 SD 1.68	58.65 SD 2.08
Weighted Healthy Life Expectancy 2021-2023	64.86 SD 2.28	63.37 SD 1.46	61.45 SD .96	59.67 SD 1.20	56.85 SD 2.15
**SOCIAL DETERMINANTS/DEPRIVATION MEASURES BY THEME**					
**EDUCATIONAL PREPAREDNESS**					
School Readiness percentage of Free School Meals 2012/13	35.42 SD 8.06	35.89 SD 7.42	33.65 SD 6.94	32.63 SD 5.88	36.90 SD 8.38
School Readiness percentage of Free School Meals 2018-19	55.93 SD 6.0	57.16 SD 5.43	56.81 SD 4.96	57.56 SD 4.85	57.74 SD 5.86
**STRUCTURAL DEPRIVATION & CRIME**					
First time entrants to the youth justice system 2010 per 100,000 pop	886.76 SD 295.11	906.22 SD 253.96	813.55 SD 198.82	1019.53 SD 377.42	943.97 SD 350.97
First time entrants to the youth justice system 2020 per 100,000 pop	161.72 SD 55.66	189.22 SD 66.74	172.01 SD 63.72	190.30 SD 87.41	182.35 SD 79.25
First time offenders per 100,00 pop 2010	478.55 SD 153.34	484.63 SD 144.15	468.92 SD 101.73	548.49 SD 172.36	532.28 SD 177.16
First time offenders per 100,000 pop 2020	159.97 SD 41.11	162.90 SD 40.75	161.90 SD 39.86	181.23 SD 51.68	176.37 SD 50.39
**ECONOMIC OPPORTUNITIES**					
16–17-year-olds not in education, employment or training (NEET) 2016 %	5.35 SD 2.01	5.53 SD 1.96	5.79 SD 1.51	6.29 SD 1.72	5.99 SD 2.02
16–17-year-olds not in education, employment or training (NEET) 2020 %	4.92 SD 2.24	5.41 SD 1.83	5.10 SD 1.58	5.59 SD 1.38	5.11 SD 1.64
Employment Gap Rate for those with long-term health condition 2013/14 %	12.87 SD 3.76	12.58 SD 3.46	15.43 SD 5.11	13.12 SD 3.34	13.58 SD 3.67
Employment Gap Rate for those with long-term health condition 2019-20 %	10.84 SD 4.09	10.69 SD 2.47	10.71 SD 4.95	11.72 SD 3.20	11.69 SD 3.76
Percentage of people in employment 2011/12	70.19 SD 5.34	70.23 SD 4.57	67.23 SD 4.18	70.07 SD 4.07	67.46 SD 5.55
Percentage of people in employment 2020-21	75.50 SD 4.19	74.62 SD 3.98	74.01 SD 3.02	75.18 SD 3.82	73.50 SD 4.20
Economic Activity 16-64 2010 percentage	76.35 SD 4.30	76.52 SD 3.26	75.15 SD 2.92	76.41 SD 3.85	74.25 SD 4.46
Economic Activity 16-64 2019 percentage	79.57 SD 3.51	78.46 SD 4.52	77.49 SD 2.92	79.15 SD 3.36	77.43 SD 4.36
**STABLE MENTAL HEALTH SERVICES**					
Adults in mental health services with stable living conditions 2011/12 %	57.54 SD 18.96	51.32 SD 17.91	62.85 SD 17.26	48.21 SD 25.32	63.46 SD 19.13
Adults in mental health services with stable living conditions 2020-21 %	61.87 SD 15.77	63.62 SD 17.08	65.92 SD 12.56	51.20 SD 20.39	57.15 SD 21.87
**PREVENTABLE VIOLENT CRIME**					
Domestic abuse-related incidents and crimes 2015/16 per 1,000	24.26 SD 7.19	25.24 SD 5.93	25.15 SD 5.47	26.47 SD 7.12	26.40 SD 6.05
Domestic abuse-related incidents and crimes 202021 per 1,000	30.98 SD 5.92	31.72 SD 8.39	33.36 SD 5.76	31.13 SD 9.55	32.21 SD 6.82
Sexual offences per 1,000 population 2010/11	0.82 SD .28	0.91 SD .29	0.92 SD .32	1.06 SD .35	0.94 SD .35
Sexual offences per 1,000 population 2020/21	2.19 SD .75	2.26 SD .73	2.57 SD .71	2.57 SD .61	2.44 SD .71
Violent offences per 1,000 population - 2010/11	12.21 SD 3.95	12.22 SD 4.99	13.13 SD 5.12	15.17 SD 5.54	13.64 SD 5.77
Violent offences per 1,000 population - 2020/21	28.60 SD 8.91	29.32 SD 8.72	33.79 SD 8.17	32.16 SD 7.76	32.29 SD 11.31
**ENVIRONMENTAL WELLBEING**					
Fuel poverty (low income, high-cost methodology) percentage 2011	10.74 SD 2.28	10.05 SD 2.01	11.63 SD 1.96	10.55 SD 2.68	11.43 SD 2.68
Fuel poverty (low income, high-cost methodology) percentage 2018	10.08 SD 2.24	9.78 SD 1.63	10.57 SD 1.50	10.12 SD 2.04	11.14 SD 2.68
Social Isolation: percentage of adult social care users 2010/11	41.77 SD 4.01	41.44 SD 4.47	41.35 SD 3.78	40.19 SD 3.25	42.35 SD 4.71
Social Isolation: percentage of adult social care users 2019/20	46.59 SD 4.60	45.43 SD 4.51	44.48 SD 4.27	45.98 SD 4.10	45.65 SD 4.30
**SEXUAL/REPRODUCTIVE HEALTH**					
Under 18s conception rate/1,000 - 2010	34.92 SD 11.49	35.03 SD 8.43	40.10 SD 10.09	37.91 SD 8.57	36.62 SD 11
Under 18s conception rate/1,000 - 2019	15.75 SD 5.51	16.33 SD 4.58	18.79 SD 6.01	17.40 SD 4.49	17.44 SD 6.97
**DEPRIVATION**					
2015 IMD - Average score	21.34 SD 7.82	21.66 SD 6.68	26.39 SD 7.47	23.65 SD 5.76	25.70 SD 8.91
2019 IMD - Average score	21.14 SD 7.81	21.74 SD 6.40	26.01 SD 8.20	23.54 SD 5.48	25.47 SD 9.01
2015 IMD - Proportion of LSOAs in most deprived 10% nationally	0.10 SD .10	0.08 SD .07	0.17 SD .13	0.09 SD.07	0.15 SD .14
2019 IMD - Proportion of LSOAs in most deprived 10% nationally	0.09 SD .11	0.09 SD .08	0.16 SD .14	0.09 SD .07	0.14 SD .15
**PREVENTABLE HEALTH OUTCOMES**					
Percentage of cancers diagnosed at stages 1 and 2 2013	54.58 SD 3.25	54.09 SD 2.47	52.29 SD 1.38	53.31 SD 2.80	54.48 SD 3.58
Percentage of cancers diagnosed at stages 1 and 2 2018	55.23 SD 2.90	54.65 SD 3.65	52.44 SD 2.94	55.36 SD 2.40	54.27 SD 3.02
Cumulative percentage aged 40-74 who received an NHS Health check 2013/14	46.15 SD 14.97	38.22 SD 14.45	42.81 SD 10.57	47.33 SD 15.94	49.05 SD 14.94
Cumulative percentage aged 40-74 who received an NHS Health check 2016/17	33.86 SD 11.12	27.73 SD 15.69	32.17 SD 9.09	34.69 SD 14.20	35.91 SD 13.01
Mortality rate from preventable causes 2009/11 per 100,00	158.32 SD 36.80	159.07 SD 33.46	188.53 SD 32.94	168.10 SD 29.44	171.18 SD 39.24
Mortality rate from preventable causes 2017/19 per 100,000	143.72 SD 35.46	147.06 SD 33.79	174.05 SD 33.79	156.10 SD 28.56	157.55 SD 40.81
Mortality rate from cardiovascular preventable 2009/11 per 100,000	36.03 SD 8.36	36.19 SD 6.42	38.97 SD 6.12	38.33 SD 8.47	39.11 SD 9.07
Mortality rate from cardiovascular preventable 2017/19 per 100,000	28.56 SD 7.47	28.88 SD 5.65	32.38 SD 5.67	31.38 SD 6.74	31.38 SD 8.12
Mortality rate from cancer preventable 2009/11 per 100,000	65.58 SD 15.43	66.67 SD 14.64	77.73 SD 14.42	66.95 SD 10.42	70.90 SD 15.46
Mortality rate from cancer preventable 2017/19 per 100,000	54.40 SD 12.73	56.47 SD 11.64	65.17 SD 13.61	58.57 SD 10.55	58.24 SD 12.42

## 2 Methods

### 2.1 Study design

We employed a retrospective (i.e., using existing, previously collected data), complexity-informed (i.e., examining interacting factors rather than single, linear effects) ecological framework (i.e., treating the data as part of a real-world system with multiple levels of influence). This took the form of a quasi-longitudinal observational study (i.e., we had multiple but somewhat varying time points in the existing data, allowing us to observe change over time without experimental manipulation), which allowed us to organised secondary data from 141 UK local authorities.

### 2.2 Dataset

Our data comprised a longitudinal place-based dataset of N = 141 English local authority administrative areas (LAs). The dataset was designed around a social determinants of health (SDH) perspective (see, Marmot et al., 2010; Marmot et al., 2020a, Marmot et al., 2020b), which has helped to inform the development of a national Public Health Outcomes Framework (Department of Health and Social Care, n.d.). Data are from the English Fingertips data repository and place-based contextual data (Ministry of Housing, Communities and Local Government, n.d., and ONS.gov.uk, n.d.). See Appendix A for detailed access to the complete dataset.

Factor Selection: Factors were chosen to move beyond the long-established focus on income deprivation and other recognized culprits to include social factors that speak directly to whether policy is preventing or mitigating inequalities. This is why, in addition to social determinants and deprivation indices, we included a full set of preventable health outcomes. We did not exclude indicators because of minor differences in reporting periods, as such variation is inherent to administrative datasets. The final N = 40 indicators reflect this balance and were agreed through investigator discussion to ensure conceptual relevance and coverage relative to Marmot et al. (2010, 2020a) – see Social Determinants, Deprivation and Health Outcomes Themes in Table 1.

Outcome: Healthy Life Expectancy (HLE) at birth, reported in rolling three-year intervals, provided us a coherent longitudinal thread from 2011 to 2023 across local authorities. We selected HLE not simply as a health metric, but as a sentinel indicator: a high-level, system-sensitive trace of population well-being that synthesizes mortality rates and self-reported health (Byrne, 2002). In contrast to conventional cross-sectional or basic pre-post designs – often ill-equipped to detect the nonlinear, feedback-driven impacts of real-world interventions – HLE trends enabled us to track the co-evolution of structure, health, and place (Castellani & Gerrits, 2024) to reflect the emergent health landscape of each locality, shaped by the contingent interplay of economic, social, and policy conditions. Crucially, HLE also anchors the interpretation of more granular indicators, situating them within broader systemic trajectories. HLE does this by measuring ‘the average number of years a person would expect to live in good health based on contemporary mortality rates and prevalence of self-reported good health …. Figures reflect the prevalence of good health and mortality among those living in an area in each time period, rather than what will be experienced throughout life among those born in the area.’ (Department of Health and Social Care, n.d.)

To create our HLE trend data from 2011 to 2023 for all 141 LAs, we combined LA scores for males and females into a single score. To do so, we generated a weighted score for each LA, for each three-year interval between 2011 and 2023, based on the relative percentage of females to males in each LA. The weighted HLE formula is as follows:

$$HLE_{weighted}=\left(HLE_{female}\;X\;\frac{\%\;female\;Pop}{100}\right)+\left(HLE_{male}\;X\;\frac{\left(100-\%\;female\;Pop\right)}{100}\right)$$

where HLE-female and HLE-male are the HLE for females and male respectively in a given LA for a given three-year time period between 2011 and 2023.

Explanatory factors: the N = 40 explanatory factors represented a range of structural, social, and policy-related conditions (e.g., indices of deprivation, school readiness, youth justice involvement, violent crime, employment, fuel poverty, and preventable mortality). These factors (organised into social determinants, deprivation indices, and preventable health outcomes shown in Table 1) were selected for their conceptual relevance to the social determinants of health and the ecological conditions shaping place-based health trajectories and policy-related health outcomes (see, Marmot et al., 2010; 2020a). As shown in Table 1, we chose these social determinants, deprivation indices and health outcomes (organised by themes) because effective proportionately universal policy and service provision should prevent or, at minimum, reduce the issue they measure.

Where possible, explanatory indicators were captured at two time points – typically near the beginning (∼2011) and end (∼2020–2023) of the period of study, though some were available only at a single time point due to data constraints. This created a *temporally staged design* (Timmons & Preacher, 2015), so the focus can be more on understanding how shifts or stasis in these upstream contextual conditions may relate to the evolution of HLE over time. We adopted a complexity-informed framework throughout, prioritizing the detection of patterned relationships, nonlinear dynamics, and contextual sensitivity over strict temporal uniformity. As Byrne (2002) argues, such an approach provides *traces* of the character of complex systems, enabling the study of system-level change using real-world, policy-relevant data across imperfect but meaningful time intervals.

### 2.3 Analyses

Our analytic strategy followed a *case-based complexity (CBC) approach*, operationalised through *COMPLEX-IT*, an R-Shiney, online, computational modelling, interdisciplinary methods platform (Schimpf & Castellani, 2020). For our study we employed a combination of machine learning, known as the self-organising map (SOM) neural net, and a SOM hierarchical clustering technique. In previous studies we have conducted, this approach has proven better than other approaches, including time-series hierarchical regression, growth mixture modelling, and latent class growth analysis (Castellani et al. 2016, 2018).

Objective: The goal was to (a) cluster the major and minor trends in our healthy life expectancy trajectories (HLE, 2011–2023) for our N = 141 English local authority administrative areas (LAs); (b) identify which configuration of N = 40 factor (organised into social determinants, deprivation indices, preventable health outcomes) helped to account for these different trends; and (c) examine these configurations in detail to develop a narrative for each cluster trend — that is, a clear account of how different combinations of factors were associated with each cluster trend and what these patterns suggest for policy and practice.

**Fig. 1:**
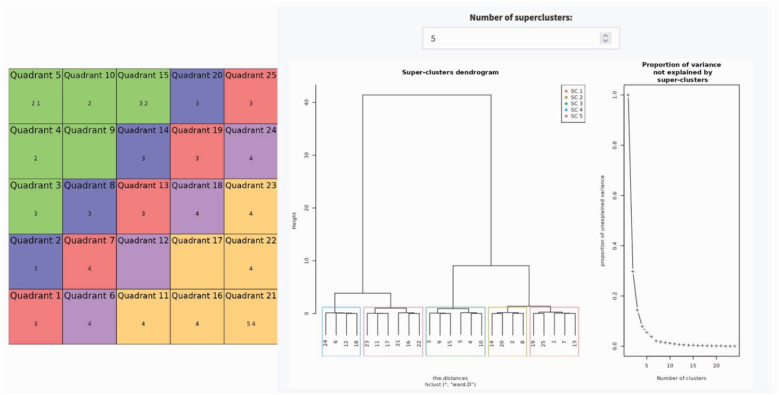
COMPLEX-IT analysis results, stage 1 and 2: SOM grid, dendrogram, and proportion of variance.

Step 1 To generate the hierarchical clusters, the SOM first requires a trained self-organising map. The strength of the SOM is that it easily handles complex nonlinear mappings of complex trend data, making it an advance on more simplistic trend fitting approaches, such as k-means, growth mixture modelling, latent class growth analysis, and generalized additive models (Castellani et al., 2014; Jain, 2010). In practice, this means we begin by running the SOM to organise the HLE trajectories for all N = 141 LAs across its grid. As shown on the left-hand side of Fig. 1, we went with a conventional 5 × 5 SOM grid, which treats the HLE data, initially, as comprised of 25 clusters called nodes, each representing a unique HLE trend. The SOM places the N = 141 LAs variously across these 25 nodes according to similar HLE profiles. The result is that across the 25 nodes (clusters) of the 5X5 SOM grid, all N = 141 LAs are located, providing the most granular cluster map of the data, N = 25 clusters.

Step 2 After the SOM arranges the N = 141 LAs on the grid across all 25 clusters (nodes), its hierarchical clustering technique helps us step back: it gathers similar nodes (clusters) into higher-level clusters, turning the detailed map into a set of clear, interpretable HLE cluster trends. The strength of SOM hierarchical clustering is that, unlike k-means, it does not impose a fixed number of clusters. Instead, it reveals how the 25 nodes cluster at successive levels of complexity. For us, this meant identifying the major clusters as well as the smaller but still policy-relevant clusters in our dataset – as clustering techniques do not, by convention, seek even distribution of cases across clusters (Jain, 2010).

Once the cluster solution is settled on, three measures are used to determine the overall level of fit. *Quantization error* measures how well the map represents the data: it is the average distance between each case and the node it is mapped to. *Topographical error* measures how well the SOM preserves the data's neighbourhood structure – specifically, the proportion of cases whose first and second best-matching units are not adjacent on the grid. Lower values for both validity tests indicate a better-fitting map. In addition, the standard deviations of the cases around each cluster are inspected to evaluate their respective homogeneity of variance. This is important for determining how well the subsequent interpretation of the HLE trends can be trusted – higher levels of homogeneity support more valid and reliable conclusions.

Step 3 With the cluster trends catalogued, we next sought to examine what configuration (i.e., profile) of N = 40 factors (i.e., social determinants, deprivation indices, preventable health outcomes) were associated with each of the major and minor HLE clusters we identified in Step 2. For each HLE cluster trend we took the average (mean) for each of our N = 40 factors for all LAs (cases) associated with each cluster, resulting in the profiles shown in Table 1.

Step 4 Finally, we used these profiles to name the clusters and interpret each trend, tracing how the underlying combination of factors helps make sense of the trajectory and its policy implications. We used standard qualitative interpretive techniques to name and explore the clusters, treating each profile as a configuration of social determinants, preventable health outcomes, and deprivation indices, and then synthesising these patterns into labels and narratives that speak directly to policy and practice.

## 3 Results

### 3.1 Clustering health inequalities across time/space

As shown on the left-hand side of Fig. 1, we went with a conventional 5 × 5 SOM grid, which treats the HLE data, initially, as comprised of 25 clusters called nodes, each representing a unique HLE trend. The mapping of the N = 141 LAs on this grid was a good fit (topographic error = 0.021; quant error = 1.44, with zero being a perfect fit) – see Methods for details.

To refine our results, we used hierarchical SOM clustering, which groups similar nodes from the SOM into higher-level patterns. The dendrogram in Fig. 1 shows the solution, which starts at the bottom treating all 25 nodes as individual clusters, ending with one cluster. The graph on the right-hand side of Fig. 1 shows the proportion of variance explained as one moves toward a single cluster, suggesting that a 5-cluster solution is useful, accounting for roughly 98–99 % of the total variance.

One could go for a simpler or more complex cluster solution, but one either loses variance accounted for or, alternatively, achieves a more granular solution but with diminishing returns. For those interested, the Appendix Dataset lists all local authorities and cluster memberships. Readers wishing to examine within-cluster variation or individual cases can treat these clusters as empirically grounded configurations — ‘tin-openers’ (Blackman et al., 2013) — that support more detailed, context-specific inquiry into local system dynamics.

For the HLE trends in Table 1, within-cluster dispersion was uniformly small – with the widest standard deviation being **1.4**–**2.2 years**, the middle trajectories (Cluster 2–4) tighter still (between **0.6**–**1.6 years**) and in several instances below **1.0 year**. Given Jain's (2009) observation that cluster analyses (even when machine learning) partition data by similarity rather than guaranteed uniformity – and can produce clusters even when no strong internal structure exists – these low dispersions indicate empirically coherent trajectories rather than outcomes arising simply from the clustering procedure.

To create the configurations for our cluster trend profiles we took the averages (mean) for each of our N = 40 factors for all LAs (cases) associated with each cluster, resulting in Table 1 – see Methods for details. Looking at the means and standard deviations for our 40 factors, variation in the indicators should be read in the context of our outcome-first design. Because the groups were formed solely on divergent HLE trajectories, differences in dispersion reflect the diversity of circumstances through which similar health-outcome patterns can emerge, rather than indicating better or worse profiles. *Clusters 1 and 3* show tighter distributions across indicators, while *Clusters 2, 4, and 5* contain a bit more internal variation; nevertheless, all five groups share broadly aligned configurations relative to their HLE trends. Rather than problematically heterogenous, these mixed clusters highlight that comparable HLE trajectories can arise through multiple configurations of deprivation, risk, and service conditions.

In what follows, we provide a description of each of the clusters, starting with the HLE trend differences and then how the configuration of indicators might account for these differences.

**Fig. 2:**
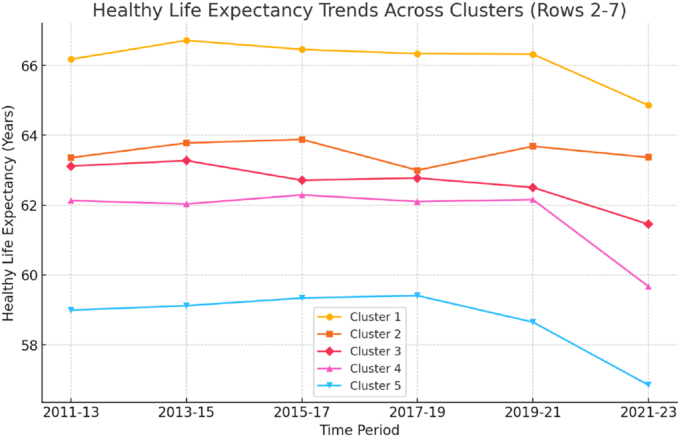
Healthy life expectancy trends across clusters.

HLE Trends: As shown in Fig. 2, across all five clusters we observe worsening HLE with increasing deprivation and growing health inequalities amongst the clusters, with the healthiest (Cluster 1) increasing its HLE gap over the least healthy (Cluster 5) from just over 7 years to 8 years. This is not surprising, as the national picture in terms of HLE is one of decline with male HLE falling by 0.8 years between 2011-13 and 2021-23 and female by 1.2 years in the same period (Raleigh, 2025). During this period health inequalities increased in England and increases in overall life expectancy and HLE stalled up until COVID-19 with subsequent sharp falls in these (Raleigh, 2025). Marmot and colleagues (2020a) highlighted the negative impact of austerity in England on inequalities and the SDH and subsequently (2020b) to this generating the context for COVID-19 to exacerbate existing health inequalities. This provides important background to, and a wider trajectory in which, the path dependencies of our clusters are situated. This is the macro context in which we seek to *trace* the emergent system dynamics at the LA level. It is also important to emphasise that the patterns are not strictly linear within a national context, revealing the importance of context-dependent social and economic policies.

**Cluster 1 –Resilient Affluence:** This cluster maintains the highest HLE, starting at 66.18 years in 2011, peaking at 66.72 years in 2013, and declining slightly to 64.86 years in 2021. The defining feature of this group is social and economic resilience, which shields it from the worst effects of structural decline. The LAs in this cluster have low crime statistics, including first-time offenders and domestic violence; the highest employment and economic activity; the lowest deprivation scores; low fuel poverty; and lowest under 18 conception rate. These LAs also have the lowest rates of mortality from preventable causes, including preventable cancers and cardiovascular disease.

**Cluster 2 – Resilient Equilibrium:** This is one of the smaller clusters of LAs (N = 13). With an HLE starting at 63.36 years (2011) and ending at 63.37 years (2021), this cluster appears relatively stable. While the absolute change is minimal, this flat trajectory stands out positively against the broader national trend of declining HLE. In this sense, the cluster has ‘held its ground’ during a period when many others have seen marked deterioration. The paradox here is that while indicators are not drastically worsening, and in fact, in many instances are some of the best scores on preventable health outcomes and economic and social wellbeing, there has been no meaningful improvement in HLE. This raises questions about the longer-term impact of policies, particularly those shaped by austerity, which may have constrained progress on the social determinants of health even where baseline conditions remain comparatively favourable.

**Cluster 3 – Holding Ground:** Another of the three smaller clusters (N = 12), HLE starts at 63.12 years (2011), improves slightly by 2013 (63.27 years), but declines to 61.45 years (2021). This cluster is one of the most intriguing, from a policy perspective. It is comprised of LAs with some of the highest rates of deprivation, including the highest proportion of lower-super-output areas in the most deprived 10 % nationally; and yet, these LAs are still doing better on HLE compared to Clusters 4 and 5, which may be due to some policy advancements, as in the case of improving employment and economic activity and reducing the unemployment gap for those with long-term health conditions, as well as gains on several preventable health outcomes. However, looking at the last few years of HLE, a trend downward may be emerging. So, while policy may be moving things in the right direction, there is still significant work to be done for these LAs, following austerity and COVID-19.

**Cluster 4 – Declining social wellbeing:** The third of the smaller clusters (N = 15), the HLE for these LAs starts at 62.13 years in 2011 and remains there, until a sharp drop to 59.67 years in 2021. The culprit may be the relationship between social wellbeing and COVID-19 and its aftermath. The LAs have significant issues: high crime; housing and employment instability for those with physical and mental health challenges; social isolation challenges; higher under 18 conception rates; and the highest rates of domestic violence and sexual offenders; combined with some of the worst rates in preventable mortality, including cardiovascular and cancer. So, while deprivation is comparatively low, there seems to be a deterioration in social, mental and physical wellbeing that may be finally manifesting itself in the last record of HLE at 59.67.

**Cluster 5 – Entrenched Disadvantage:** The lowest HLE across all groups, starting at 58.99 years (2011), peaking slightly at 59.41 years (2017), and dropping to 56.85 years (2021). Like Cluster 3, the LAs in this cluster struggle significantly with deprivation, including the second highest proportion of LSOAs in the most deprived 10 % nationally. Unlike Cluster 3, however, these LAs are not holding ground. Employment, including for those with long-term health conditions, is a major challenge, along with the lowest scores on economic activity. Domestic violence, sexual offences, unstable living conditions for those with mental health challenges are all significant challenges for these LAs – which seem to go hand-in-hand with some of the worst mortality rates from preventable causes. The LAs in this cluster are struggling and it is strongly linked to deprivation and socioeconomic disadvantage.

## 4 Discussion

The methodological and conceptual contributions of this paper mark a significant departure from conventional approaches to health inequalities. Traditional variable-driven models, though effective at identifying general correlations, have struggled to capture the emergent, nonlinear, and context-dependent nature of health disparities. In turn, configurational approaches such as qualitative comparative analysis (QCA) have been far less common and cross-sectional (e.g., Blackman et al., 2011, 2013). By integrating case-based complexity with computational modelling, this study advances the field beyond static, reductionist frameworks that have historically dominated public health and policy analysis (Byrne, 2005; Salway & Green, 2017; Schrecker, 2021; Wistow et al., 2015), offering a more rigorous and empirically grounded alternative, one that preserves the specificity of place while identifying broader structural patterns that shape health outcomes over time. It also provides an empirical anchor showing how place-based health trajectories emerge from distinct configurations of social, economic and institutional conditions. The clusters serve as ‘traces’ of underlying system dynamics, revealing, for example, where local systems are resilient, where they are stagnating, and where they are structurally locked into decline.

At the core of this methodological advance are two insights. First, health inequalities are not simply a function of individual-level risk factors or even singular social determinants but rather the product of historically contingent, self-organising social systems. The application of COMPLEX-IT within a case-based complexity framework enables a more nuanced interrogation of these systems. Unlike traditional clustering methods, which often impose artificial categorizations based on static variables, this approach identifies emergent groupings rooted in real-world trajectories of change (Schimpf & Castellani, 2022). As a result, rather than reducing complexity to a single gradient of deprivation, this study uncovered distinct clusters of health inequality, each requiring a different theoretical and policy response. This requires a fundamental shift in how we conceptualize the problem itself: place-based health inequalities are not merely different degrees of the same phenomenon but represent qualitatively different social configurations with distinct causal mechanisms and policy needs. Second, novel insights can be gained by exploring those health and social factors that function as proxies for demonstrating if place-based policies are mitigating/exacerbating inequalities.

### 4.1 Rethinking and calibrating policy

Interventions in complex social systems must be grounded in an understanding of local system dynamics, using interdisciplinary evidence to design context-sensitive approaches that work with, rather than against, the system to achieve sustainable change (Moore et al., 2019). The social gradient in the SDH also must be recognized as shaping a corresponding gradient in the tractability of policy solutions to address health inequities. Wistow (2022) contends that effective policy must be calibrated to the scale and character of population needs, their relationship to socio-economic and spatial inequalities, and the uneven capacity of multi-level governance systems to respond accordingly. Using an interdisciplinary framing, we consider policy implications across the clusters.

**Cluster 1 – Policy causal asymmetry:** There are two policy takeaways from this cluster. First, it reinforces the value of effective policy. Not to say that gains cannot still be made. But, by comparison, things are going comparatively well for these LAs. A complexity-based intervention could focus on sustaining resilience, while introducing additional interventions, for example, workplace health programmes (Sorensen et al., 2021), chronic disease prevention (Airhihenbuwa et al., 2021) improved air quality (Castellani et al., 2022) and climate change adaptation and resilience (Climate Change Committee, 2025) to anticipate and pre-empt emergent risks before they lead to decline.

The second policy takeaway is causal asymmetry: each cluster has a distinct causal configuration, so interventions effective in Cluster 1 may not translate to Clusters 2–5. This creates a policy paradox of success for Cluster 1 — progress must be actively maintained, yet these same measures for Cluster 1's success may not translate to more deprived clusters. In fact, the progressive success of Cluster 1 may exacerbate existing inequalities in other Clusters, as areas with better health have less population need and may have greater relative resourcing and capacity to calibrate policy innovations into effective practice. This is the value of having multiple clusters: they allow us to see how different configurations of deprivation and resilience produce divergent health trajectories, which require different policy interventions. This shifts policy thinking away from singular, universal interventions toward adaptive, networked solutions tailored to how places behave in each context.

**Cluster 2 – Building upon an equilibrium:** In complexity terms, this cluster comprises LAs operating within a relatively stable equilibrium, one in which health outcomes neither markedly improve nor deteriorate. In the context of nationally declining HLE, this equilibrium suggests the presence of reinforcing feedback loops that sustain current outcomes. There is a dual policy challenge: understanding and strengthening these stabilising mechanisms; and introducing adaptive interventions that can shift systems toward improved trajectories without undermining underlying structural resilience. This calls for a shift to complexity-informed approaches that examine how communities organise for health and how they use those insights to scale interventions on the social determinants (Matheson et al., 2024). Policies should build on the factors that have maintained relative stability while incorporating catalytic measures, such as economic, social, and health initiatives that are already contributing incrementally to improvement. Lessons from Cluster 1 may offer valuable insights, though any transfer of strategy must be sensitive to causal asymmetries and local context.

**Cluster 3 – Systematically drive what works and improve what isn't working:** Relative to causal asymmetry, what makes this cluster significant from a policy standpoint is that, unlike Clusters 4 and 5, which are not doing well, these LAs, despite some of the worst deprivation rates, are holding ground. Areas of positive policy impact can be found in relation to employment and economic activity, in particular. Cluster 3 appears to be a policy success; the priority is to scale effective practices, providing a template for supporting struggling areas without resorting to one-size-fits-all, ineffective approaches. Policymakers aiming to reduce health inequalities in Clusters 4 and 5 can prioritise learning from the relative success of Cluster 3, rather than directly replicating policies from Clusters 1 or 2. Cluster 3 also demonstrates that progress is possible even under conditions of significant deprivation, offering more contextually relevant insights for similarly disadvantaged areas. Still, a genuinely adaptive, multi-level complex systems response must account for spillover effects, since policies that work in advantaged clusters may, in other contexts, amplify rather than reduce inequalities – as illustrated in our review of Cluster 1. In this respect, national policy should carefully assess the effects of strategies across the social gradient, identifying and mitigating the risk of reinforcing structural disparities.

**Cluster 4 – Economic deprivation is only part of the problem:** Cluster 4 demonstrates the inadequacy of simple deprivation policy models in explaining variations in HLE. While deprivation remains a core determinant in this Cluster, its effects manifest through nonlinear social inequality and poverty pathways, which appear to have had a social impact, leading to lower HLE outcomes across time. Addressing these challenges requires moving beyond reactive or siloed approaches toward more integrated, multi-layered strategies. Areas in this cluster may benefit from adopting new governance approaches, including (1) empowering local government to address health inequalities, (2) supporting community engagement and civil society, (3) increasing health and care funding proportionate to need, (4) building and retaining a health and care workforce capable of delivering equity, strengthening the focus on SDH in health systems and policy platforms, and (5) monitoring SDH from a health equity standpoint (WHO, 2025). Policies must be socially as well as economically responsive, with active, not passive, interventions that address interdependent challenges simultaneously. Instead of targeting singular risk factors (e.g., criminal justice reform), policy should focus on multi-layered interventions, such as embedding economic revitalization within community safety policies, to simultaneously stabilize the system from multiple entry points. In addition, as Readhead and Lynch (2024) demonstrate discourses of geographical ‘unfairness’ can obscure the structural roots of place-based health inequalities, highlighting the need to investigate how cultural values around health, place, and fairness shape both public understanding and policy responses.

**Cluster 5 – Transformative, non-incremental interventions:** This is the most structurally locked-in Cluster, characterised by entrenched socio-economic deprivation and significant health disadvantage. Complexity science argues that addressing deep-rooted socio-spatial inequalities demands transformative, system-wide change focused on the structural causes of socio-economic inequality through a whole-systems approach (Coburn, 2009; Navarro, 2009; Rod et al., 2023; WHO, 2025). In the UK context, these disparities are historically ingrained and require a fundamental shift in both policy approach and resource allocation (Martin et al., 2021; Telford & Wistow, 2022). Disruptive, whole-system interventions can break entrenched patterns of disadvantage and overcome the path dependency that characterises these places. Wistow (2022) argues that this necessitates a more redistributive political economy and structural reforms capable of recalibrating complex systems and challenging their underlying logics. For example, policies that expand income-based resources can help reduce place-based health inequalities in areas left behind by a spatially imbalanced economy (Simpson et al., 2025), but such efforts demand sustained coordination and investment given capitalism's inherent tendency toward uneven spatial development (Hudson, 2022; Jones, 2019; Streeck, 2016). In addition, the development of ‘health first’ approaches to address worklessness and enhance productivity, through the creation of high-quality, secure employment for individuals with health conditions linked to targeted support and incentives for these individuals and their employers (Bambra et al., 2025).

**Clusters 1**–**5:** Taken together, the clusters trace a configurational, rather than linear, movement down a place-based social gradient in health, with each step from Cluster 1 to Cluster 5 reflecting progressively weaker structural conditions and declining system capacity for sustaining wellbeing. By identifying these gradient-aligned configurations, our analysis provides a realistic foundation for designing adaptive, multi-level policies that work with, rather than against, the complex systems through which health inequalities emerge.

## 5 Limitations and future research

Despite our advances, our approach has three major limitations. First, as with any clustering technique, while the SOM offers a statistically valid partition, different algorithms or subsets of our 40 indicators might yield slightly different groupings. Future work should test these alternatives using both qualitative and quantitative approaches. To support this, we have made our data publicly available so others can examine or challenge our cluster solutions.

Second, Step 3 (linking clusters to indicators) was descriptive rather than inferential. We chose to accept this limitation for very specific reasons: we resisted clustering directly on the N = 40 social-determinant variables or searching for ‘driving factors’ for our HLE cluster trends, as doing so would reproduce organised common sense: areas already similar on deprivation would cluster together, offering little insight beyond organised common sense as to why the HLE trajectories diverged across cluster trends. We ran this exact analysis post-hoc and found, indeed, our results were as expected, yielding little innovative insights. By clustering outcomes first, however, and examining configurations through comparative case-based analyses second, we were able to identify patterned differences within and across the five clusters that move beyond predictable associations or the search for the most salient factors. Future research could use our dataset to compare a different approach to ours.

Third, this study used only one component of the broader CBC toolkit: trajectory clustering and configurational profiling (Castellani & Gerrits, 2024). CBC is, by design, a multimethod framework, and future research could extend this work through complementary approaches such as participatory systems mapping, scenario simulation, or digital storytelling to deepen within-case understanding and support policy calibration. These methods align with the core aim of CBC: to trace how configurations operate within lived systems and to engage policymakers and communities in interpreting and acting on those traces. Developing such a multi-method rubric is beyond the scope of the present paper, but our findings provide a foundation for such work.

Finally, as a reminder, our indicator set was chosen not to restate the well-known link between deprivation and poor health, but to include factors that show whether policy is mitigating entrenched inequalities. Preventable health outcomes, alongside social determinants and deprivation indices, offered a practical way to read system performance rather than structural disadvantage alone. Although England is data-rich, the same approach can be applied elsewhere using a smaller set of proxy indicators that capture preventable outcomes and the core social conditions policy is meant to address. Even a leaner configuration can support a case-based understanding of how inequalities persist and where policy leverage points may lie.

## 6 Conclusion

To address health inequalities effectively, we must adopt a policy paradigm rooted in complexity-informed, multi-level governance that embraces the dynamic, emergent nature of social systems. A CBC perspective frames health disparities not as isolated or static outcomes, but as manifestations of interdependent social, economic, and institutional trajectories. Drawing on Byrne's (2002) notion of “traces of complex systems”, the patterns we observe (whether through clustering or narrative) should be understood as empirical imprints of underlying system dynamics. These traces offer a means to explore how structural forces shape health outcomes in place, and how policy might intervene meaningfully within those configurations.

Such an approach demands policy calibration that is sensitive to both local specificity and systemic interconnectivity, recognising that place-based interventions can generate unintended consequences across clusters if not carefully aligned. Rather than applying one-size-fits-all solutions, a CBC-informed framework supports a shift towards adaptive, proportionate universalism embedded within national strategies that respond to diverse local realities while maintaining coherence across scales. This is not only a more realistic way of conceptualising the policy challenge, but a necessary step toward avoiding the reproduction of inequalities under the guise of improvement.

## CRediT authorship contribution statement

**Brian Castellani:** Writing – review & editing, Writing – original draft, Visualization, Validation, Software, Methodology, Formal analysis. **Jonathan Wistow:** Writing – review & editing, Writing – original draft, Methodology, Formal analysis, Data curation, Conceptualization.

## Declaration of generative AI and AI-assisted technologies in the writing process

During the preparation of this work the authors used ChatGPT in order to improve the readability of a few parts of the paper. During and after the use of this tool, the authors reviewed and edited the content as needed and take full responsibility for the content of the published article.

## Declaration of competing interest

The authors declare that they have no known competing financial interests or personal relationships that could have appeared to influence the work reported in this paper.

## Appendix A

Go to https://art-sciencefactory.com/OnlineDataSet.html for the complete dataset to run in COMPLEX-IT or other computational/statistical software. It contains the SOM NODE ID (1 through 25) and SOM CLUSTER ID (1 through 5) for each of the N = 141 LAs, along with their profile across all N = 40 explanatory variables. There are also a few notes on running the dataset in COMPLEX-IT.

## Footnotes

1 There are currently 153 upper tier local authorities in England. Our dataset excludes those with missing data but has near complete England-wide coverage. English upper tier local authorities (including metropolitan boroughs, unitary authorities and county councils) form the strategic level of local government responsible for services such as public health, education, transport, and social care. They vary in population size and geography, ranging from densely populated urban authorities to large rural counties. These differences create distinct demographic profiles and local governance challenges, in which upper tier authorities are a diverse but central arm of local government and democracy and for understanding place-based policy and public health in England.

## References

[bib1] Acheson D. (1998).

[bib2] Airhihenbuwa C.O., Tseng T.S., Sutton V.D., Price L. (2021). Global perspectives on improving chronic disease prevention and management in diverse settings. Preventing Chronic Disease.

[bib3] Bambra C., McNamara C., Munford L., Wickham S. (2025). To get Britain working we need to get Britain healthy. BMJ.

[bib4] Barbrook-Johnson P., Penn A. (2022).

[bib6] Black D. (1980).

[bib7] Blackman T., Wistow J., Byrne D. (2011). A qualitative comparative analysis of factors associated with narrowing health inequalities in England. Social Science & Medicine.

[bib8] Blackman T., Wistow J., Byrne D. (2013). Using qualitative comparative analysis to understand complex policy problems. Evaluation.

[bib9] Byrne D. (2002).

[bib10] Byrne D. (2005). Complexity, configurations and cases. Theory, Culture & Society.

[bib12] Byrne D., Callaghan G. (2023).

[bib13] Byrne D., Ragin C. (2009).

[bib14] Cabinet Office (2010).

[bib15] Castellani B., Barbrook-Johnson P., Schimpf C. (2019). Case-based methods and agent-based modelling: Bridging the divide to leverage their combined strengths. International Journal of Social Research Methodology.

[bib16] Castellani B., Bartington S., Wistow J., Heckels N., Ellison A., van Tongeren M., Arnold S., Barbrook-Johnson P., Bicket M., Pope F., Russ T., Clarke C., Pirani M., Schwannauer M., Vieno M., Turnbull R., Gilbert N., Reis S. (2022). Mitigating the impact of air pollution on dementia and brain health: Setting the policy agenda. Environmental Research.

[bib17] Castellani B. (2018). Exploring comorbid depression and physical health trajectories: A case-based computational modelling approach. Journal of Evaluation in Clinical Practice.

[bib18] Castellani B., Gerrits L. (2024).

[bib19] Castellani B., Rajaram R., Buckwalter J.G., Ball M., Hafferty F. (2015).

[bib20] Castellani B., Rajaram R., Gunn J., Griffiths F. (2016). Cases, clusters, densities: Modeling the nonlinear dynamics of complex health trajectories. Complexity.

[bib21] Castellani B., Schimpf C., Wistow J., Caden C., Agarwal J., Barbrook-Johnson P. (2025). Case-based systems mapping: Advancing a multimethod approach to social complexity. International Journal of Social Research Methodology.

[bib22] Childs (2024).

[bib23] Climate Change Committee (2025).

[bib24] Coburn D., Panitch L., Leys C. (2009). Morbid symptoms: Health under capitalism.

[bib26] Department for Health and Social Care (n.d.) Fingertips: Public health profiles, https://fingertips.phe.org.uk/profile/public-health-outcomes-framework (last accessed 17/9/25).

[bib27] Doyal L., with Pennell I. (1979).

[bib28] Gerrits L., Chang R.A., Pagliarin S. (2021). Case-based complexity: Within-case time variation and temporal casing. Complexity, Governance & Networks.

[bib30] Haim L., Klaber B., Sowemimo A., Marmot M. (2024). NHS and the whole of society must act on socil determinants of health for a healthier future. BMJ.

[bib31] Hudson R. (2022). ‘Levelling up’ in post-Brexit United Kingdom: Economic realism or political opportunism?. Local Economy.

[bib32] Hunter D., Littlejohns P., Weale A. (2024). Public health is in crisis, but it can be fixed. BMJ.

[bib33] Jain A.K. (2010). Data clustering: 50 years beyond K-means. Pattern Recognition Letters.

[bib34] Jones M. (2019).

[bib35] Lansley S. (2021).

[bib36] Lynch J. (2017). Reframing inequality? The health inequalities turn as a dangerous frame shift. Journal of Public Health.

[bib37] Marmot M. (2015).

[bib38] Marmot M., Allen J., Boyce T., Goldblatt P., Morrison J. (2020).

[bib39] Marmot M., Allen J., Boyce T., Goldblatt P., Morrison J. (2020).

[bib40] Marmot Review (2010).

[bib41] Martin R., Gardiner B., Pike A., Sunley P., Tyler P. (2021).

[bib42] Matheson A., Wehipeihana N., Gray R., Walton M., Uia T., Lindberg K., Shanthakumar M., Irurzun Lopez M., Reidy J., Firestone R., Ellison-Loschmann L. (2024). Building a systems-thinking community workforce to scale action on determinants of health in New Zealand. Health & Place.

[bib43] Ministry of Housing Communities and Local Government (n.d.) English indices of deprivation, https://www.gov.uk/guidance/english-indices-of-deprivation-2019-mapping-resources (last accessed 17/9/25).

[bib44] Moore G., Evans R., Hawkins J., Littlecott H., Melendez-Torres G., Bonell C., Murphy S. (2019). From complex social interventions to interventions in complex social systems: Future directions and unresolved questions for intervention development and evaluation. Evaluation.

[bib45] Navarro V. (2009). What we mean by social determinants of health. Global Health Promotion.

[bib46] Office for National Statistics (n.d.) Economy https://www.ons.gov.uk/economy (last accessed 17/9/25).

[bib47] Pearce J.R. (2018). Complexity and uncertainty in geography of health research: Incorporating life-course perspectives. Annals of the Association of American Geographers.

[bib48] Raleigh V. (2025). What is happening to life expectancy in England?. The King’s Fund.

[bib49] Readhead G., Lynch R. (2024). The unfairness of place: A cultural history of the UK's ‘postcode lottery’. Health & Place.

[bib50] Rod N., Broadbent A., Rod M., Russo F., Arah O., Stronks K. (2023). Complexity in epidemiology and public health. Addressing complex health problems through a mix of epidemiologic methods and data. Epidemiology.

[bib51] Salway S., Green J. (2017). Towards a critical complex systems approach to public health. Critical Public Health.

[bib52] Scambler G., Scambler S. (2015). Theorizing health inequalities: The untapped potential of dialectical critical realism. Social Theory & Health.

[bib54] Schimpf C., Castellani B. (2020). COMPLEX-IT: A case-based modelling and scenario simulation platform for social inquiry. Journal of Open Research Software.

[bib55] Schimpf C., Castellani B. (2024). Approachable modelling and smart methods: A new methods field of study. International Journal of Social Research Methodology.

[bib56] Schrecker T. (2017). Was Mackenbach right? Towards a practical political science of redistribution and health inequalities. Health & Place.

[bib57] Schrecker T. (2021). What is critical about critical public health? Focus on health inequalities. Critical Public Health.

[bib58] Simpson, J., Albani, V., Munford, L., & Bambra, C. (Accepted/2025. Left behind? A longitudinal, ecological study of regional deprivation amplification and life expectancy growth in in England, 2004 to 2020. Health & Place. 10.1016/j.healthplace.2025.103478 PMC7619329 40339500

[bib59] Sorensen G., Dennerlein J.T., Peters S.E., Sabbath E.L., Kelly E.L., Wagner G.R. (2021). The future of research on work, safety, health and wellbeing: A guiding conceptual framework. Social Science & Medicine.

[bib60] Streeck W. (2016).

[bib61] Telford L., Wistow J. (2022).

[bib62] Timmons A., Preacher J. (2015). The importance of temporal design: How do measurement intervals affect the accuracy and efficiency of parameter estimates in longitudinal research?. Psychological Methods.

[bib63] UK2070 Commission (2020).

[bib64] United Nations (2019).

[bib65] Wistow J. (2022).

[bib66] Wistow J., with Blackman T., Byrne D., Wistow G. (2015).

[bib67] World Health Organization (n.d.) Social determinants of health: https://www.who.int/health-topics/social-determinants-of-health#tab=tab_1 (last accessed 30/4/24).

[bib68] World Health Organization (2008).

[bib69] World Health Organization (2025).

